# RES3/417: The Development of a Clinical Trial Web Site: a proposal for a model

**DOI:** 10.2196/jmir.1.suppl1.e79

**Published:** 1999-09-19

**Authors:** E Santoro, E Nicolis, MG Franzosi

**Affiliations:** ^1^Istituto di Ricerche Farmacologiche Mario Negri, MilanoItaly

**Keywords:** Clinical Trial, Internet, World Wide Web, Trial Web Site

## Abstract

**Introduction:**

The Internet and the World Wide Web have been recently introduced in the management of some aspects of large scale clinical trials. We have developed a model for a clinical trial Web site in order to enhance communication among people involved in a clinical trial, to disseminate clinical trials information, and to decentralise some trials activities.

**Methods:**

One section of the Web site should include general medical information such as summary of the background, aim, design of the ongoing trial and results of similar trials. A directory of investigators, members committees, sponsors and monitors (with their e-mail address) could also help people in improving communication. Another section should include centre based summaries of the trial: randomised patients, follow-up schedule, patients lost to follow-up, outstanding case report forms and queries, and statistics on centres quality are some examples. An additional section should be dealing with the latest trial news: trial newsletter, planned meetings, congresses presentations, archive of frequently asked questions on the application of the trial protocol. Patient randomisation/registration and remote data entry are two additional components of a clinical data management system that could be included in the site. In this case database access rules and security should be implemented. Other Web based applications are related with the supply of study material (protocol, case report forms, informed consent), the management of mailing lists for the investigators, the publishing of sub-analyses results.

**Discussion:**

Although some problems still exist (Internet connection limitations, security, lack of common standard for client software, personnel training), the Internet and the clinical trial Web sites could in the few years transform the way a clinical trial is conducted.

